# Prognostic significance of fludeoxyglucose positron emission tomography delta radiomics following bridging therapy in patients with large B-cell lymphoma undergoing CAR T-cell therapy

**DOI:** 10.3389/fimmu.2024.1419788

**Published:** 2024-10-01

**Authors:** Colton Ladbury, Claire Hao, William Tyler Watkins, Sagus Sampath, Jeffrey Wong, Arya Amini, Karen Sokolov, Jekwon Yeh, Karine A. Al Feghali, Dorine de Jong, Arjun Maniyedath, Shervin Shirvani, Liana Nikolaenko, Matthew Mei, Alex Herrera, Leslie Popplewell, Lihua Elizabeth Budde, Savita Dandapani

**Affiliations:** ^1^ Department of Radiation Oncology, City of Hope National Medical Center, Duarte, CA, United States; ^2^ RefleXion Medical, Inc., Hayward, CA, United States; ^3^ Department of Hematology and Hematopoietic Cell Transplantation, City of Hope National Medical Center, Duarte, CA, United States

**Keywords:** large B-cell lymphoma (LBCL), delta radiomics, bridging radiation, chimeric antigen receptor T-cell (CAR T), positron emission tomography

## Abstract

**Purpose/objective(s):**

Bridging radiation therapy (bRT) is increasingly being utilized prior to chimeric antigen receptor (CAR) T-cell therapy for large B-cell lymphoma (LBCL). It is unknown how the extent of cytoreduction during bRT impacts outcomes.

**Materials/methods:**

We retrospectively reviewed patients with LBCL treated with bRT followed by CAR T-cell therapy. Metabolic tumor volume (MTV), maximum standardized uptake value (SUV_max_), SUV_mean_, and total lesion glycolysis (TLG) were extracted from F18-fluorodeoxyglucose positron emission tomography (PET) scans acquired prior to bRT and between completion of bRT and CAR T-cell infusion. Delta radiomics based on changes of these values were then calculated. The association between delta radiomics and oncologic outcomes [progression-free survival (PFS), freedom from distant progression (FFDP), and local control (LC)] were then examined.

**Results:**

Thirty-three sites across 23 patients with LBCL were irradiated. All metabolically active disease was treated in 10 patients. Following bRT, median overall decreases (including unirradiated sites) in MTV, SUV_max_, SUV_mean_, and TLG were 22.2 cc (63.1%), 8.9 (36.8%), 3.4 (31.1%), and 297.9 cc (75.8%), respectively. Median decreases in MTV, SUV_max_, SUV_mean_, and TLG in irradiated sites were 15.6 cc (91.1%), 17.0 (74.6%), 6.8 (55.3%), and 157.0 cc (94.6%), respectively. Median follow-up was 15.2 months. A decrease in SUV_max_ of at least 54% was associated with improved PFS (24-month PFS: 83.3% vs. 28.1%; p = 0.037) and FFDP (24-month FFDP: 100% vs. 62.4%; p < 0.001). A decrease in MTV of at least 90% was associated with improved FFDP (24-month FFDP: 100% vs. 62.4%; p < 0.001). LC was improved in sites with decreases in SUV_max_ of at least 71% (24-month LC: 100% vs. 72.7%; p < 0.001). Decreases of MTV by at least 90% (100% vs. 53.3%; p = 0.038) and TLG by at least 95% (100% vs. 56.3%; p = 0.067) were associated with an improved complete response rate.

**Conclusion:**

bRT led to substantial reductions in MTV, SUV_max_, SUV_mean_, and TLG. The relative extent of these decreases correlated with improved outcomes after CAR T-cell infusion. Prospective cohorts should validate the value of interim PET following bRT for quantifying changes in disease burden and associated prognosis.

## Introduction

Chimeric antigen receptor (CAR) T-cell therapy has become an important treatment option for patients with relapsed/refractory large B-cell lymphoma (LCBL), with three Food and Drug Administration–approved autologous CD19-directed therapies [axicabtagene ciloleucel (axi-cel), lisocabtagene maraleucel (liso-cel), and tisagenlecleucel (tisa-cel)] being available (1–3). Due to the length of time that it takes to manufacture CAR T cells following leukapheresis, many patients are given so-called bridging therapy (BT) in the interval between leukapheresis and lymphodepletion/CAR T-cell infusion for treatment of symptomatic, life-threatening, bulky, or persistent disease. BT can be administered in the form of systemic therapy (bST), radiation therapy (bRT), or both [as combined modality therapy (bCMT)].

Published studies suggest that bRT is commonly used and is safe and effective (4–8), although not curative, serving mainly to palliate or cytoreduce. High response rates to bRT in chemo-refractory cases (9), may lead to better CAR T cell therapy outcomes by decreasing disease burden which inversely correlates with higher efficacy (10–16). However, there are limited data on how the overall changes in disease during bRT cytoreduction, quantified using positron emission tomography (PET) radiomic metrics, might impact outcomes. This can partially be attributed to the fact that obtaining PET imaging in the interval between completion of bRT and lymphodepletion is not standard. However, PET/CT has been established as the standard imaging modality for assessing response and predicting outcomes following CAR T-cell therapy (17). Therefore, if radiomic changes could be quantified, then it might provide insight into whether bRT can enhance outcomes beyond its initial cytoreductive effect. This is crucial, as two-thirds of CAR T-cell recipients experience progression with few subsequent salvage treatment options (1–3, 18).

To this end, we performed a single-institution retrospective analysis of patients who underwent bRT and had PET imaging available both before bRT and in the interval between completion of bRT and before lymphodepletion/CAR T-cell infusion. PET radiomic metrics were extracted for each scan, which permitted calculation of delta radiomics based on their temporal changes. We then examined the impact of the delta radiomics on disease outcomes following CAR T-cell infusion.

## Materials and methods

### Patient selection

Following institutional review board approval, we conducted a retrospective analysis of patients with R/R LBCL who underwent leukapheresis and infusion of a commercially available CD19 CAR T-cell product between April 2019 and December 2022. The data cutoff date for follow-up was 24 January 2023. During this period, consecutive patients who met inclusion criteria were identified for inclusion in this analysis. Patients with diffuse LBCL (DLBCL), high-grade B-cell lymphoma, primary mediastinal B-cell lymphoma (PMBCL), and transformed follicular lymphoma (tFL) were included. Patients were required to have received bRT between leukapheresis and lymphodepletion and have PET imaging available from before the start of bRT and after completion or bRT but before CAR T-cell infusion. Due to difficulty in quantifying disease in the central nervous system (CNS) due to background PET signal, patients with CNS disease were excluded from the analyses. Patients were staged using the Ann Arbor system based on disease extent at time of bRT.

### Bridging therapy and CAR T-cell therapy

All patients received bRT or bCMT prior to lymphodepleting chemotherapy and within 60 days of CAR T infusion. bCMT included treatment with bRT and bST in the form of chemotherapy and/or targeted therapy. bST was administered at the discretion of the treating hematologist. bRT treatment site and dose were administered at the discretion of the treating radiation oncologist. Radiation doses were converted to equivalent dose in 2-Gy fractions (EQD2) using an α/β of 10. Patients who received low-dose steroids for side-effect management during radiation were not considered to have received bCMT. bRT that encompassed all metabolically active tumor at the time of simulation was considered comprehensive, whereas bRT that only targeted certain sites of disease was considered focal. Comprehensive bRT was administered whenever it was felt to be feasible to encompass all disease in the radiation field without excessive toxicity. Lymphodepletion chemotherapy consisted of either cyclophosphamide (300–500 mg/m^2^) and fludarabine (30 mg/m^2^) administered on days −5, −4, and −3 or bendamustine (90 mg/m^2^) administered on days −5 and −4. Dose adjustments for organ dysfunction were per treating physician discretion. CAR T-cell treatment consisted of a single infusion of axi-cel, tisa-cel, or liso-cel on day 0.

### Laboratory values

Lab values including white blood count (WBC), absolute lymphocyte count (ALC), absolute neutrophil count (ANC), platelets (PLT), and lactate dehydrogenase (LDH) from the most recent lab values before bRT and the most recent lab values after bRT but before lymphodepletion were extracted from each patient’s charts. A simplified Endothelial Activation and Stress Index (EASIX) score (LDH/PLT) was then calculated.

### PET metric definition

**Figure 1:**
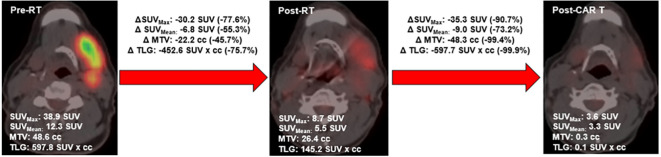
Example PET delta-radiomic example in patient with favorable response to bRT.

On each PET scan, the following metrics were extracted: metabolic tumor volume (MTV), maximum standardized uptake value (SUV_max_), SUV_mean_, and total lesion glycolysis (TLG). MTV was defined as the sum of all volumes within the PET-CT image with an uptake ≥1.5 × mean SUV + 2 standard deviations (SD) of a 3-cm^3^ volume of interest placed in the normal region of the right lobe of the liver, consistent with the previously described PERCIST method (19, 20). MTV was contoured using autosegmentation algorithms using Velocity v4.0, with final volumes modified to exclude regions of physiologic uptake. SUV_max_ was defined as the maximum SUV measurement within the MTV. SUV_mean_ was defined as the mean SUV measurement within the MTV. To calculate TLG, the SUV_mean_ was multiplied by the MTV. These values were extracted for each patient’s entire body and for individual treated lesions. Maximum dimension was also extracted, which was defined as the maximum size of active disease in any plane. Individual sites were delineated as follows: nodal sites were defined as distinct nodal regions (defined by Ann Arbor staging), and extranodal sites were defined as distinct masses with clear planes of separation from other tumors on CT based on prior approaches (21). Delta radiomics of changes of these values from the pre-bRT scan to the post-bRT scan were then calculated. An example of this analysis is shown in **Figure 1**.

### Statistical analysis

Descriptive statistics of patient and BT details were tabulated. The paired Wilcoxon signed-ranked test and one-tailed Fisher’s exact test were used to compare continuous and categorical variables, respectively, from before and after bRT. The association between delta-radiomic metrics or radiation dose on disease response, progression-free survival (PFS), overall survival (OS), local control (LC), and freedom from distant progression (FFDP) was assessed. Disease response evaluations were conducted using PET-CT, based on the Lugano classification (22). PET-CT assessments were obtained at baseline prior to bRT, following bRT but before CAR T-cell infusion, at 1 month after infusion, and 3 months after infusion. Additional PET-CTs were obtained on an individual patient basis based off response, symptoms, and concern for progression. Disease response evaluations were tabulated following bRT, 30 days after CAR T-cell infusion, and at the time of best response. PFS was defined as any disease progression, relapse, or death resulting from any cause. OS was defined as death resulting from any cause. LC was defined as absence of disease progression within the planning target volume (PTV) of treated lesions. FFDP was defined as any disease progression or relapse outside the planning target volume (PTV) of treated lesions. All outcomes were calculated from the date of CAR T-cell infusion. PFS and OS were evaluated using Kaplan–Meier survival analyses. LC and FFDP were evaluated using Fine-Gray competing risk survival analyses, with death as a competing risk. Optimal cut points were determined using maximally selected log-rank. Spearman correlation coefficients were used to assess relationships within disease burden (PET metrics and LDH) measurements. Linear regression was used to evaluate the relationship between radiation dose and delta-radiomic metrics. The cutoff for data analysis was 14 March 2023. All confidence intervals reported are 95%. As a sensitivity analysis to ensure that results were not confounded by patients receiving bCMT, the above analyses were repeated excluding those patients. Statistical analyses were performed using R version 4.2.2 (The R Foundation, Indianapolis, IN, USA).

## Results

### Patient and radiation characteristics

Full patient and radiation characteristics are summarized in **Table 1**. A total of 23 patients with LBCL with 33 irradiated sites were reviewed. Median age at time of CAR T-cell infusion was 65.6 years. Eleven (47.8%) patients had stage III/IV disease. Patients received a median of three prior treatment lines. The head and neck region was the most commonly irradiated site (48.5% of sites). A total of 24.2% of sites were extranodal and 30.3% were bulky (>7.5 cm). The most common fractionation schedule was 3 Gy × 10 fractions, with median dose of 25 Gy and median EQD2 of 26 Gy. Ten (43.5%) patients received comprehensive radiation encompassing all sites of disease. Four (17.4%) patients received concurrent systemic therapy with bRT. Median time between pre-bRT PET and the first fraction of bRT was 31 days (interquartile range, 23.5–54). Median time between completion of bRT and interim PET was 9 days (interquartile range, 5–18).

**Table 1:** Patient and treatment characteristics.

Patient characteristics	
Characteristic	N (%)
Age, years, median (range)	65.6 (21.5–85.6)
Sex	
Female	11 (47.8%)
Male	12 (52.2%)
Race and ethnicity	
Asian	6 (26.1%)
Hispanic White	5 (21.7%)
Non-Hispanic White	12 (52.2%)
ECOG performance status	
0	11 (47.8%)
1	10 (43.5%)
2	2 (8.7%)
DLBCL	18 (78.3%)
PMBCL	2 (8.7%)
tFL	2 (8.7%)
tMZL	1 (4.3%)
Double/triple hit	4 (17.4%)
Double expresser	4 (17.4%)
Cell of origin	
GCB	12 (52.2%)
Non-GCB	9 (39.1%)
Unknown	2 (8.7%)
Stage	
I	5 (21.7%)
II	7 (30.4%)
III	3 (13%)
IV	8 (34.8%)
IPI	
0	3 (13%)
1	10 (43.5%)
2	8 (34.8%)
3	2 (8.7%)
Prior treatment lines, median (range)	3 (1–5)
RT characteristics	
Characteristic	N (%)
Treated site	
Axilla	2 (6.1%)
Chest wall	2 (6.1%)
Extremity	5 (15.2%)
Head and neck	16 (48.5%)
Pelvis	1 (3%)
Retroperitoneum	3 (9.1%)
Spine	1 (3%)
Thorax	3 (9.1%)
Extranodal site	8 (24.2%)
Bulky site	10 (30.3%)
RT dose, Gy, median (range)	25 (14 - 44)
RT fractions, median (range)	10 (5 - 22)
EQD2, Gy, median (range)	26 (14 - 44)
Fractionation regimens	
2 Gy × 10	6 (18.2%)
2.5 Gy × 10	5 (15.2%)
2.5 Gy × 15	3 (9.1%)
3 Gy × 10	8 (24.2%)
3 Gy × 5	3 (9.1%)
4 Gy × 5	2 (6.1%)
Comprehensive RT fields	10 (43.5%)
Concurrent systemic therapy	4 (17.4%)
Pembrolizumab	1 (4.3%)
R-BENDA-POLA	2 (8.7%)
R-GEMOX	1 (4.3%)

ECOG, Eastern Cooperative Oncology Group; DLBCL, diffuse large B-cell lymphoma; PMBCL, primary mediastinal B-cell lymphoma; tFL, transformed follicular lymphoma; tMZL, transformed marginal zone lymphoma; GCB, germinal center B-cell; IPI, international prognostic index; RT, radiation therapy; EQD2, equivalent dose in 2-Gy fractions; R-BENDA-POLA, rituximab bendamustine polatuzumab vedotin; R-GEMOX, rituximab gemcitabine oxaliplatin.

### Lab values

Changes in lab values are summarized in **Supplementary Figure 1**. Median change in LDH was −37 U/L (p < 0.01). Following bRT, there were significant decreases in all hematologic parameters except simplified EASIX. This included decreases in platelet count (−39 K/µL, p < 0.001), WBC (−1.2 K/µL, p < 0.001), ALC (K/µL, p < 0.001), and ANC (−0.5 K/µL p < 0.01). Notably, with the exception of ALC, which had a baseline of 0.8 K/µL and decreased to 0.5 K/µL, these reductions were not to values below the lower limit of normal. No high-grade toxicity was reflected in the lab tests.

### Delta-radiomic metrics

**Figure 2:**
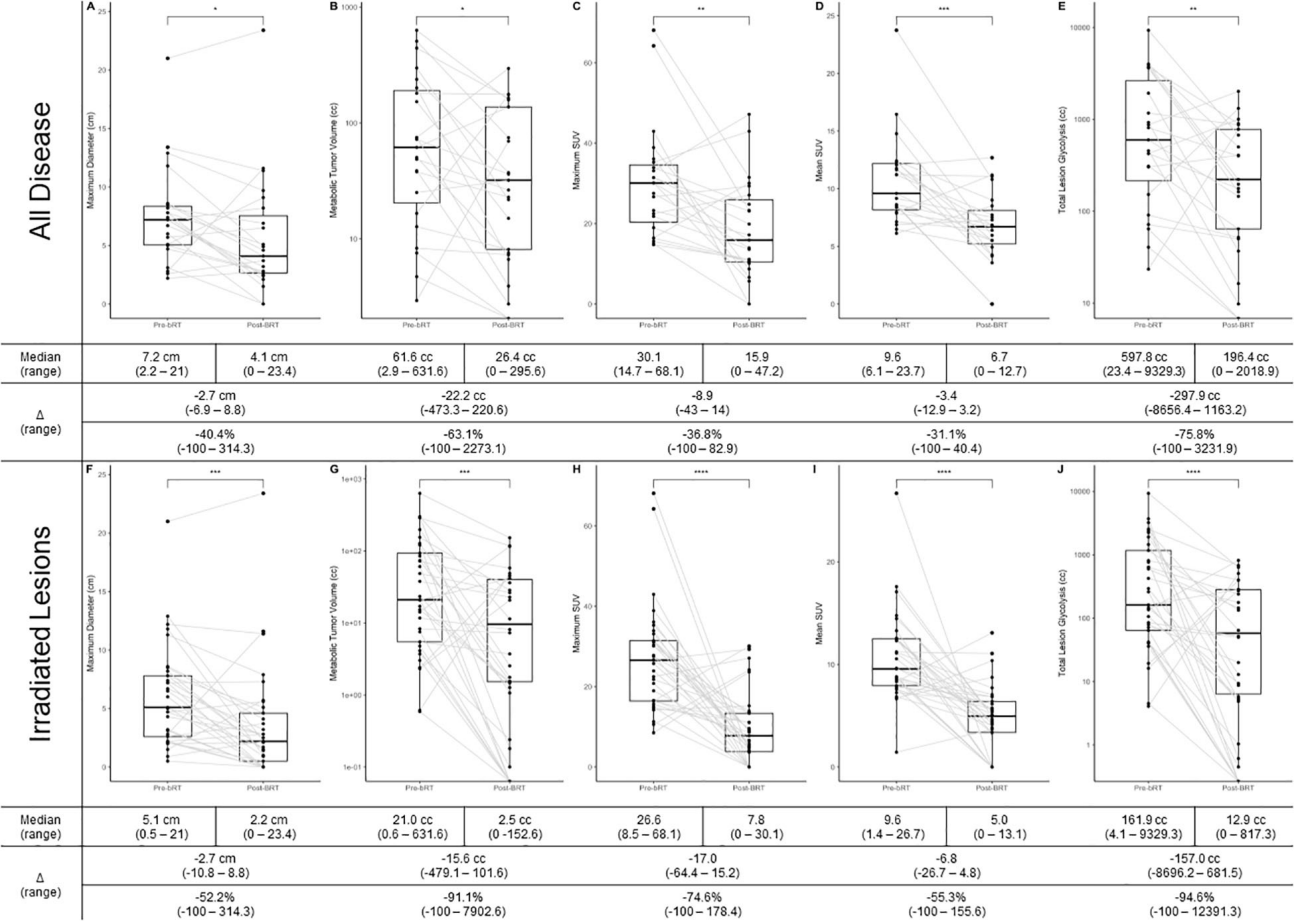
**(A)** Change in maximum dimension in all disease; **(B)** Change in MTV in all disease; **(C)** Change in SUVmax in all disease; **(D)** Change in SUVmean in all disease; **(E)** Change in TLG in all disease; **(F)** Change in maximum dimension in irradiated disease; **(G)** Change in MTV in irradiate disease; **(H)** Change in SUVmax in irradiated disease; **(I)** Change in SUVmean in irradiated disease; **(J)** Change in TLG in irradiated disease. * p<0.05, ** p<0.01, *** p<0.001, **** p<0.0001;

Changes in PET metrics are summarized in **Figure 2**. bRT was associated with significant decreases in all disease metrics, with the greatest effect in irradiated lesions. The maximum disease diameter decreased by 2.7 cm (40.4%, p < 0.05) and 2.7 cm (52.2%, p < 0.001) in irradiated lesions. MTV decreased by 22.2 cc (63.1%, p < 0.05) and 15.6 cc (91.1%, p < 0.05) in irradiated lesions. SUV_max_ decreased by 8.9 (36.8%, p < 0.01) and 17.0 (74.6%, p < 0.0001) in irradiated lesions. SUV_mean_ decreased by 3.4 (31.1%, p < 0.001) and 6.8 (55.3%, p < 0.0001) in irradiated lesions. TLG decreased by 297.7 cc (75.8%, p < 0.01) and 157.0 cc (94.6%, p < 0.0001) in irradiated lesions.

In the four patients who received bCMT, three had overall reductions in MTV following bRT [median, −194.3 cc (range, −433.0–158.4); −74.2% (−63.1–953.9)]. Seven lesions were treated in these patients, and five had reductions in MTV following bRT [median −126.0 cc (range, −288.4–101.6); −81.5% (−98.7–1789.1)]. All four patients received focal bRT and so had unirradiated disease. Three patients had an increase in MTV in areas that were unirradiated [median, 7.4 cc (range, −129.0–16.8); 7672.9% (−67.6–462,566.7)].

**Figure 3:**
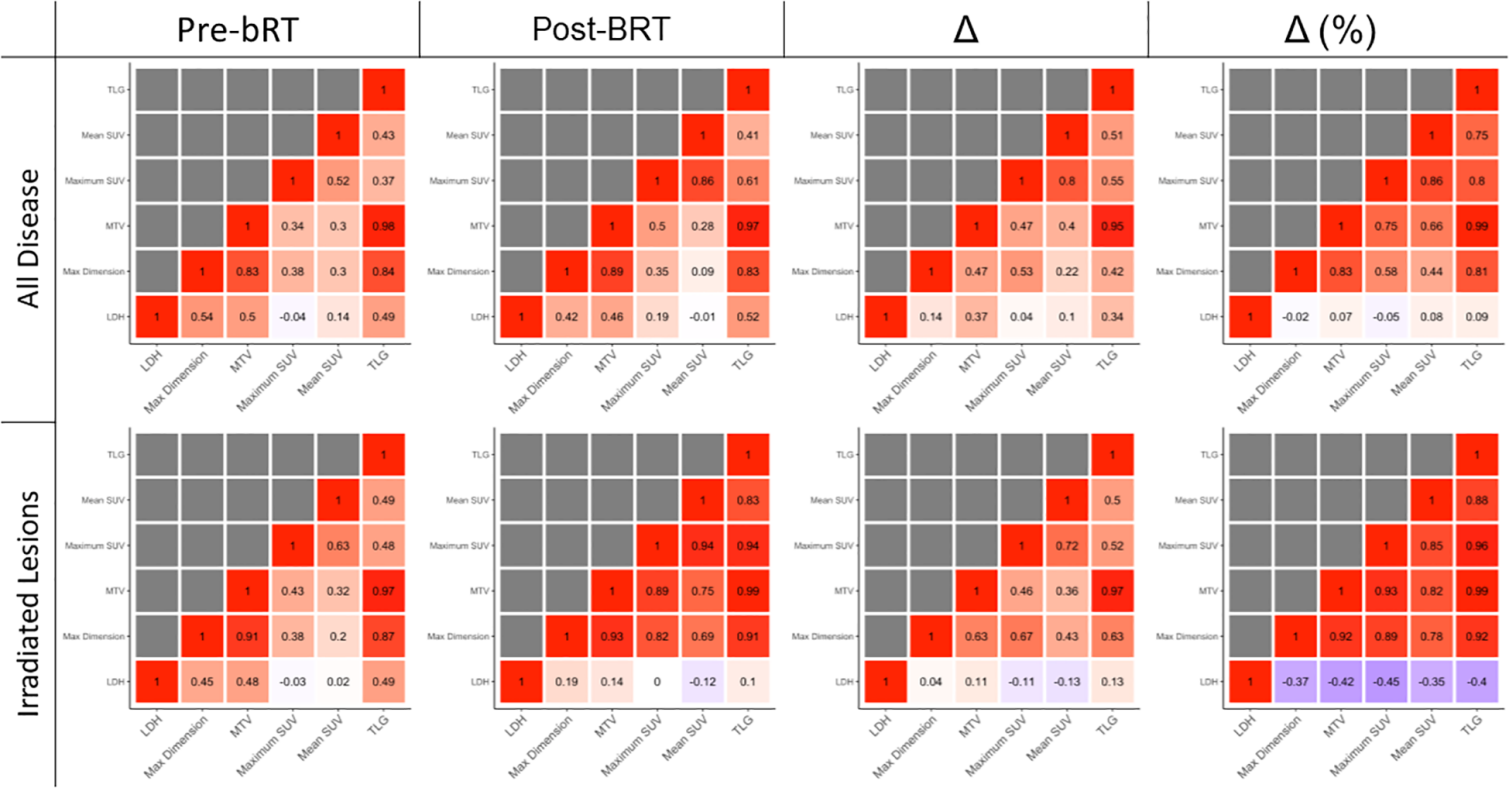
Correlations between PET radiomic metrics.

Correlations between disease burden metrics are shown in **Figure 3**. Changes in LDH, whether absolute or relative, were not strongly correlated with changes in any PET metric.

Correlations were greatest in irradiated lesions measured in percentage changes. In irradiated lesions, percentage change in SUV_max_ was strongly correlated with percentage change in TLG (R = 0.96) and MTV (R = 0.93). In irradiated disease, maximum dimension was also strongly correlated with TLG (R = 0.92) and MTV (R = 0.92). In all disease, SUV_max_ correlated with MTV (R = 0.83) and TLG (R = 0.80). In all disease, maximum dimension was also correlated with MTV (R = 0.75) and TLG (R = 0.81).

### Dose–response relationship

The impact of radiation dose, measured in EQD2, on change in PET metrics is shown in **Supplementary Figure 2**. Increasing dose was associated with greater absolute changes in SUV_mean_ (p = 0.008) and relative changes in SUV_mean_ (p = 0.01) and TLG (p = 0.072). For all PET metrics, the linear relationship between EQD2 and changes was negative.

### Hemato-oncologic outcomes

**Figure 4:**
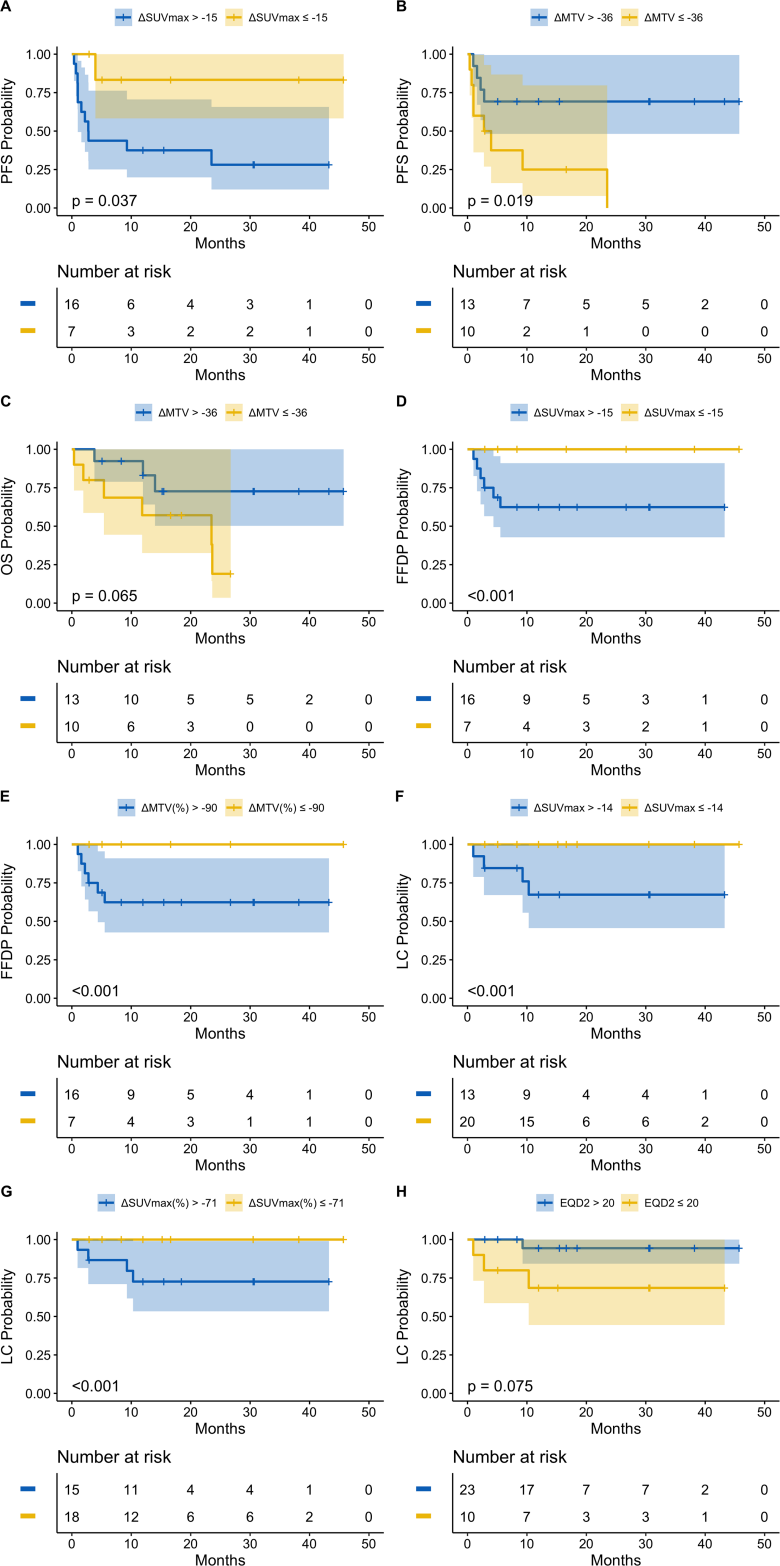
**(A)** PFS stratified by ΔSUVmax; **(B)** PFS stratified by ΔMTV; **(C)** OS stratified by ΔMTV; **(D)** FFDP stratified by ΔSUVmax; **(E)** FFDP stratified by ΔMTV%; **(F)** LC stratified by ΔSUVmax; **(G)** LC stratified by ΔSUVmax%; **(H)** LC stratified by EQD2.

Median follow-up for all patients was 15.2 months (range, 0.4–45.7). Prognostic thresholds for PET metric changes, determined by maximally selected log-rank, and associated impact on 12- and 24-month survival are summarized in **Table 2**. Selected survival curves are shown in **Figure 4**. All survival curves can be found in **Supplementary Figures 3**-**7**. A decrease in SUV_max_ of at least 54% was associated with improved PFS (24-month PFS: 83.3% vs. 28.1%; p = 0.037) and FFDP (24-month FFDP: 100% vs. 62.4%; p < 0.001). Paradoxically, a decrease in MTV of at least 36 was associated with inferior PFS (24-month PFS: 0% vs. 69.2%; p = 0.019) and OS (24-month OS: 19.0% vs. 72.7%; p = 0.065). However, relative changes in MTV of at least 90% were associated with improved FFDP (24-month FFDP: 100% vs. 62.4%; p < 0.001). LC was improved in sites with absolute or relative decreases in SUV_max_ of at least 14 (24-month LC: 100% vs. 67.3%; p < 0.001) or 71% (24-month LC: 100% vs. 72.7%; p < 0.001), respectively. LC was also improved in lesions that received an EQD2 of greater than 20 Gy (24-month LC: 94.4% vs. 68.6%; p = 0.075).

**Table 2:** Survival statistics for identified delta-radiomic PET metrics.

Metric	12-month PFS	24-month PFS	p
ΔSUV_max_ (>−15 vs. ≤−15)	37.5% (19.9–70.6) vs. 83.3% (58.3–100)	28.1% (12–65.7) vs. 83.3% (58.3–100)	**0.037**
ΔSUV_mean_ (>−3 vs. ≤−3)	36.4% (16.6–79.5) vs. 61.7% (37.6–100)	36.4% (16.6–79.5) vs. 46.3% (21.8–98.2)	0.16
ΔMTV, cc (>−36 vs. ≤−36)	69.2% (48.2–99.5) vs. 25% (7.8–79.7)	69.2% (48.2–99.5) vs. 0% (0–0)	**0.019**
ΔTLG, cc (>−910 ≤−910)	60% (39.7–90.7) vs. 31.2% (10.2–95.5)	60% (39.7–90.7) vs. 0% (0–0)	0.12
ΔSUV_max_, % (>−54 vs. ≤−54)	37.5% (19.9–70.6) vs. 83.3% (58.3–100)	28.1% (12–65.7) vs. 83.3% (58.3–100)	**0.037**
ΔSUV_mean_, % (>−55 vs. ≤−55)	40% (21.5–74.3) vs. 72.9% (46.8–100)	40% (21.5–74.3) vs. 48.6% (19.5–100)	0.31
ΔMTV, % (>−90 vs. ≤−90)	43.8% (25.1–76.3) vs. 55.6% (23.1–100)	35% (17.2–71) vs. 55.6% (23.1–100)	0.16
ΔTLG, % (>−95 vs. ≤−95)	43.8% (25.1–76.3) vs. 55.6% (23.1–100)	35% (17.2–71) vs. 55.6% (23.1–100)	0.16
ΔEQD2, Gy (>20 vs. ≤20)	58.3% (37.4–90.9) vs. 37.5% (15.3–91.7)	46.7% (25–87.1) vs. 37.5% (15.3–91.7)	0.44
Metric	12-Month OS	24-Month OS	p
ΔSUV_max_ (>−15 vs. ≤−15)	62.5% (42.8–91.4) vs. 100% (100–100)	44.4% (23.8–83) vs. 66.7% (30–100)	0.19
ΔSUV_mean_ (>−3 vs. ≤−3)	54.5% (31.8–93.6) vs. 90.9% (75.4–100)	43.6% (21.8–87.4) vs. 60.6% (33.4–100)	0.13
ΔMTV, cc (>−36 vs. ≤−36)	83.1% (64.1–100) vs. 57.1% (32.6–100)	72.7% (50.3–100) vs. 19% (3.5–100)	*0.065*
ΔTLG, cc (>−910 ≤−910)	70.5% (49.8–99.7) vs. 75% (50.3–100)	61.7% (39.9–95.3) vs. 25% (4.8–100)	0.42
ΔSUV_max_, % (>−54 vs. ≤−54)	62.5% (42.8–91.4) vs. 100% (100–100)	44.4% (23.8–83) vs. 66.7% (30–100)	0.19
ΔSUV_mean_, % (>−55 vs. ≤−55)	60% (39.7–90.7) vs. 100% (100–100)	52.5% (32.2–85.6) vs. 50% (18.8–100)	0.35
ΔMTV, % (>−90 vs. ≤−90)	62.5% (42.8–91.4) vs. 100% (100–100)	44.4% (23.8–83) vs. 66.7% (30–100)	0.19
ΔTLG, % (>−95 vs. ≤−95)	62.5% (42.8–91.4) vs. 100% (100–100)	44.4% (23.8–83) vs. 66.7% (30–100)	0.19
ΔEQD2, Gy (>20 vs. ≤20)	72.2% (52.4–99.6) vs. 71.4% (44.7–100)	60.2% (37.2–97.4) vs. 26.8% (5.6–100)	0.57
Metric	12-month FFDP	24-month FFDP	p
ΔSUV_max_ (>−15 vs. ≤−15)	62.4% (42.8–91) vs. 100% (100–100)	62.4% (42.8–91) vs. 100% (100–100)	**<0.001**
ΔSUV_mean_ (>−3 vs. ≤−3)	54.3% (32–92.4) vs. 91.7% (77.3–100)	54.3% (32–92.4) vs. 91.7% (77.3–100)	*0.076*
ΔMTV, cc (>−36 vs. ≤−36)	76.9% (57.1–100) vs. 67.2% (43–100)	76.9% (57.1–100) vs. 67.2% (43–100)	0.731
ΔTLG, cc (>−910 ≤−910)	66% (45.7–95.4) vs. 87.5% (67.3–100)	66% (45.7–95.4) vs. 87.5% (67.3–100)	0.393
ΔSUV_max_, % (>−54 vs. ≤−54)	62.4% (42.8–91) vs. 100% (100–100)	62.4% (42.8–91) vs. 100% (100–100)	**<0.001**
ΔSUV_mean_, % (>−55 vs. ≤−55)	59.9% (39.8–90.2) vs. 100% (100–100)	59.9% (39.8–90.2) vs. 100% (100–100)	**<0.001**
ΔMTV, % (>−90 vs. ≤−90)	62.4% (42.8–91) vs. 100% (100–100)	62.4% (42.8–91) vs. 100% (100–100)	**<0.001**
ΔTLG, % (>−95 vs. ≤−95)	62.4% (42.8–91) vs. 100% (100–100)	62.4% (42.8–91) vs. 100% (100–100)	**<0.001**
ΔEQD2, Gy (>20 vs. ≤20)	79.4% (61.2–100) vs. 60% (33.1–100)	79.4% (61.2–100) vs. 60% (33.1–100)	0.371
Metric	12-month LC	24-month LC	p
ΔSUV_max_ (>−14 vs. ≤−14)	67.3% (45.6–99.4) vs. 100% (100–100)	67.3% (45.6–99.4) vs. 100% (100–100)	**<0.001**
ΔSUV_mean_ (>−7 vs. ≤−7)	74.2% (55.7–99) vs. 100% (100–100)	74.2% (55.7–99) vs. 100% (100–100)	**<0.001**
ΔMTV, cc (>−35 vs. ≤−35)	95% (85.9–100) vs. 74.3% (53.3–100)	95% (85.9–100) vs. 74.3% (53.3–100)	0.185
ΔTLG, cc (>−400 ≤−400)	95% (85.9–100) vs. 74.3% (53.3–100)	95% (85.9–100) vs. 74.3% (53.3–100)	0.185
ΔSUV_max_, % (>−71 vs. ≤−71)	72.7% (53.4–99) vs. 100% (100–100)	72.7% (53.4–99) vs. 100% (100–100)	**<0.001**
ΔSUV_mean_, % (>−55 vs. ≤−55)	74.2% (55.7–99) vs. 100% (100–100)	74.2% (55.7–99) vs. 100% (100–100)	**<0.001**
ΔMTV, % (>−59 vs. ≤−59)	74.9% (54.1–100) vs. 93.3% (81.5–100)	74.9% (54.1–100) vs. 93.3% (81.5–100)	0.169
ΔTLG, % (>−73 vs. ≤−73)	70% (46.7–100) vs. 94.1% (83.4–100)	70% (46.7–100) vs. 94.1% (83.4–100)	*0.098*
ΔEQD2, Gy (>20 vs. ≤20)	94.4% (84.3–100) vs. 68.6% (44.5–100)	94.4% (84.3–100) vs. 68.6% (44.5–100)	*0.075*

PFS, progression-free survival; OS, overall survival; FFDP, freedom from distant progression; LC, local control; SUV, standardized uptake value; MTV, metabolic tumor volume; TLG, total lesion glycolysis; EQD2, equivalent dose in 2-Gy fractions. Bolded - p<0.05; Italics p<0.10

Response rates, stratified by thresholds previously identified using the maximally selected log-rank, are summarized in **Table 3**. The delta-radiomic thresholds were most predictive of best treatment response relative to post-bRT response of 30-day response. Decreases in SUV_max_ of at least 15 (100% vs. 56.3%; p = 0.067) and SUV_mean_ (91.7% vs. 40%; p = 0.015) of at least 3 were associated with improved CRR. Relative decreases of MTV by at least 90% (100% vs. 53.3%; p = 0.038) and TLG by at least 95% (100% vs. 56.3%; p = 0.067) were also associated with improved CRR.

**Table 3:** Delta-radiomic PET metric predictors of treatment response.

Post-bRT response				
Metric	CRR	p	ORR	p
ΔSUV_max_ (>−15 vs. ≤−15)	1 (5.9%) vs. 1 (16.7%)	0.462	12 (70.6%) vs. 6 (100%)	0.184
ΔSUV_mean_ (>−3 vs. ≤−3)	0 (0%) vs. 2 (16.7%)	0.261	7 (63.6%) vs. 11 (91.7%)	0.131
ΔMTV, cc (>−36 vs. ≤−36)	2 (15.4%) vs. 0 (0%)	0.308	8 (61.5%) vs. 10 (100%)	0.038
ΔTLG, cc (>−910 ≤−910)	2 (12.5%) vs. 0 (0%)	0.474	11 (68.8%) vs. 7 (100%)	0.13
ΔSUV_max_, % (>−54 vs. ≤−54)	0 (0%) vs. 2 (28.6%)	0.083	11 (68.8%) vs. 7 (100%)	0.13
ΔSUV_mean_, % (>−55 vs. ≤−55)	0 (0%) vs. 2 (28.6%)	0.083	11 (68.8%) vs. 7 (100%)	0.13
ΔMTV, % (>−90 vs. ≤−90)	0 (0%) vs. 2 (28.6%)	0.083	11 (68.8%) vs. 7 (100%)	0.13
ΔTLG, % (>−95 vs. ≤−95)	0 (0%) vs. 2 (33.3%)	0.059	12 (70.6%) vs. 6 (100%)	0.184
ΔEQD2, Gy (>20 vs. ≤20)	1 (6.7%) vs. 1 (12.5%)	0.889	12 (80%) vs. 6 (75%)	0.792
30-day response				
Metric	CRR	p	ORR	p
ΔSUV_max_ (>−15 vs. ≤−15)	6 (37.5%) vs. 6 (100%)	0.012	14 (87.5%) vs. 6 (100%)	0.519
ΔSUV_mean_ (>−3 vs. ≤−3)	3 (30%) vs. 9 (75%)	0.046	9 (90%) vs. 11 (91.7%)	0.805
ΔMTV, cc (>−36 vs. ≤−36)	8 (61.5%) vs. 4 (44.4%)	0.361	12 (92.3%) vs. 8 (88.9%)	0.844
ΔTLG, cc (>−910 ≤−910)	9 (56.3%) vs. 3 (50%)	0.771	15 (93.8%) vs. 5 (83.3%)	0.481
ΔSUV_max_, % (>−54 vs. ≤−54)	6 (40%) vs. 6 (85.7%)	0.059	13 (86.7%) vs. 7 (100%)	0.455
ΔSUV_mean_, % (>−55 vs. ≤−55)	6 (40%) vs. 6 (85.7%)	0.059	14 (93.3%) vs. 6 (85.7%)	0.909
ΔMTV, % (>−90 vs. ≤−90)	6 (40%) vs. 6 (85.7%)	0.059	13 (86.7%) vs. 7 (100%)	0.455
ΔTLG, % (>−95 vs. ≤−95)	7 (43.8%) vs. 5 (83.3%)	0.119	14 (87.5%) vs. 6 (100%)	0.519
ΔEQD2, Gy (>20 vs. ≤20)	9 (64.3%) vs. 3 (37.5%)	0.221	13 (92.9%) vs. 7 (87.5%)	0.879
Best response				
Metric	CRR	p	ORR	p
ΔSUV_max_ (>−15 vs. ≤−15)	9 (56.3%) vs. 6 (100%)	0.067	14 (87.5%) vs. 6 (100%)	0.52
ΔSUV_mean_ (>−3 vs. ≤−3)	4 (40%) vs. 11 (91.7%)	0.015	9 (90%) vs. 11 (91.7%)	0.905
ΔMTV, cc (>−36 vs. ≤−36)	10 (76.9%) vs. 5 (55.6%)	0.276	12 (92.3%) vs. 8 (88.9%)	0.844
ΔTLG, cc (>−910 ≤−910)	11 (68.8%) vs. 4 (66.7%)	0.733	15 (93.8%) vs. 5 (83.3%)	0.481
ΔSUV_max_, % (>−54 vs. ≤−54)	9 (60%) vs. 6 (85.7%)	0.243	13 (86.7%) vs. 7 (100%)	0.455
ΔSUV_mean_, % (>−55 vs. ≤−55)	9 (60%) vs. 6 (85.7%)	0.243	14 (93.3%) vs. 6 (85.7%)	0.909
ΔMTV, % (>−90 vs. ≤−90)	8 (53.3%) vs. 7 (100%)	0.038	13 (86.7%) vs. 7 (100%)	0.454
ΔTLG, % (>−95 vs. ≤−95)	9 (56.3%) vs. 6 (100%)	0.067	14 (87.5%) vs. 6 (100%)	0.519
ΔEQD2, Gy (>20 vs. ≤20)	11 (78.6%) vs. 4 (50%)	0.182	13 (92.9%) vs. 7 (87.5%)	0.879

PET, positron emission tomography; bRT, bridging radiation; CRR, complete response rate; ORR, objective response rate; LC, local control; SUV, standardized uptake value; MTV, metabolic tumor volume; TLG, total lesion glycolysis; EQD2, equivalent dose in 2-Gy fractions.

### Sensitivity analysis

Following exclusion of patients who received bCMT, the above analyses were repeated and produced consistent findings with similar changes in PET metrics, overall dose responses, and hemato-oncologic outcomes.

## Discussion

Here, we report a retrospective analysis of the prognostic significance of PET delta radiomics, corresponding to cytoreduction, resulting from bRT prior to standard of care CD19 CAR T-cell therapy for R/R LBCL. In our patient cohort, there were substantial reductions in SUV_max_, SUV_mean_, MTV, and TLG, which is particularly notable given that the follow-up scan was obtained a median of 9 days (range, 2–41) following completion of bRT, when bRT may not have achieved its maximal response effect and post-bRT inflammation may have artificially increased PET metric measurements. We also found that overall extent of cytoreduction, measured in relative changes in PET metrics, can predict multiple outcomes following CAR T-cell infusion. This adds to existing data that bRT may confer a local additive benefit to the systemic effects of CAR T-cell therapy by cytoreducing lymphoma volume. Of note, unsurprisingly, PET metrics were generally found to be strongly correlated. The utility of this correlation is that, where it is not possible to determine either MTV, TLG, or SUV_mean_ (due to these metrics requiring segmentation of active disease), total dimension and SUVmax, which are readily found in most radiology reports, may be useful surrogates. Our data support the use of relative changes, rather than absolute changes, in PET metrics as small absolute changes in MTV were still associated with improved PFS and OS. Although this finding seems paradoxical at first glance, this is likely because patients with less disease burden going into bRT (which is associated with an improved prognosis) are limited in the absolute extent of PET metric reduction that is possible. Subsequently, when patients are stratified by an optimized threshold, these lower-risk patients are combined with patients with more disease that responded poorly, thereby compensating for the outcomes in those higher-risk patients. This problem is remedied by using relative changes.

Overall disease burden, quantified by MTV, is a known prognostic factor for patients undergoing CD19 CAR T-cell therapy for R/R LBCL based on several analyses (10–16). PET metrics are also predictive of patterns of failure. In an analysis of 63 patients and 469 lesions by Figura et al., lesions that were ≥5 cm in diameter had a maximum standardized uptake value ≥10, or those that were extranodal were at an increased risk of local failure (21). Optimally BT such as bRT addresses higher risk disease and produces improved LC of treated lesions and overall PFS. Although limited by sample size, in our cohort, the LC rates were highest in lesions that would not have met high-risk criteria or that no longer met high-risk criteria following bRT.

The relevance of cytoreduction following bRT was first evaluated in a retrospective series by Hubbeling et al. (23) This study analyzed outcomes, including patterns of failure, in 41 patients (33 with DLBCL, 7 with mantle cell lymphoma, and 1 with Burkitt lymphoma) who underwent bRT. Thirty-two patients had pre-bRT and post-bRT PET imaging available. In these patients, there was an 84% in-field objective response rates, although overall 56% of patients progressed during bRT. Following bRT, there were significant decreases in SUV_max_, and maximum disease diameter, with a greater effect when only treated sites were evaluated. Overall, there was no significant change in MTV, although there was a significant decrease in treated lesions. This study identified three risk groups: patients were considered poor risk if they had an MTV >16.4 cc post-bRT, good risk if their pre- and post-bRT MTVs were ≤16.4 cc, and converted risk if their pre-bRT MTV was >16.4 cc and post-bRT MTV improved to ≤16.4 cc. Although sample sizes limited the ability to perform log-rank testing, patients with good risk and converted risk had similar OS and PFS, which were improved relative to patients with poor risk. Thus, it is possible that use of bRT as a local cytoreductive therapy, in combination with CAR T-cell as a systemic therapy, could have a synergistic effect, whether it is via simply decreasing the targeted lymphoma volume for the CAR T cells or through an immune-stimulatory effect (24, 25). Our data add to these findings, wherein not only does absolute amount of disease going into CAR T-cell treatment matter but also the extent of the change over the course of BT.

Delta radiomics with bST were evaluated by Sesques et al. in a study of 72 patients of whom nearly all received bST without concurrent bRT (16). They too found that tumor control between leukapheresis and lymphodepletion was a better predictor of PFS than pre-lymphodepletion absolute MTV values. Patients with changes in MTV <300% and TLG <420% had significant longer PFS, indicating the bST’s role in disease stabilization rather than reduction. This finding may be skewed by selection bias, as bST is often administered for more extensive disease. Additionally, there could also be contributions from patients’ prior heavily pre-treated chemo-refractory disease with 48% having more than four prior lines of treatment, suggesting a distinct impact from bST. If disease kinetics and extent of cytoreduction are indeed prognostic as suggested by several studies (12, 16), then interim PET following completion of bRT or other forms of BT could inform whether to intensify BT for those with suboptimal response. However, such a decision must be weighed against the risk of greater toxicity and potential delays in CAR T-cell therapy. Alternatively, monitoring of PET response over the course of bRT using a linear accelerator with integrated PET imaging could facilitate a risk adaptive approach on a per-fraction basis without extending overall treatment time (26). This has the potential to address a significant clinical need and may represent a major improvement over previous options; CAR T-cell therapy only produces a durable remission in about a third of patients (1–3, 18). Existing retrospective data on using bRT to augment these outcomes are favorable, although this needs to be validated prospectively and an adaptive approach would be met with logistical challenges including interim PET not yet being standard of care.

Research on the prognostic value of PET metrics has mainly focused on MTV, which is excellent for measuring tumor volume but can be challenging to calculate without specialized software, which could hinder its clinical adoption. We find that SUV_max_ and maximum lesion diameter, which are both routinely reported on radiology reports, correlated with MTV and may serve as effective clinical surrogate metrics, particularly given that relative changes in SUV_max_ was associated with various outcomes.

Our dose–response analyses support selective dose escalation. With improved LC observed in patients receiving EQD2 higher than 20 Gy. Remarkably, no local failures occurred at an EQD2 higher than 32.5 Gy. Further, increasing dose was associated with greater reduction in PET metrics. However, favorable outcomes were still observed in several patients that received lower doses. This is consistent with findings by Sim et al., where no failures occurred when treated with a dose ≥37.5 Gy or EQD2 ≥39 Gy, but median EQD2 was 24 Gy and only 16.9% of lesions failed overall (27). Of these failures, 77.8% occurred in lesions greater than 50 cc. This suggests that lower doses may be sufficient for lesions without adverse features, such as increased size, SUV, or extranodal location (21), whereas higher dose might benefit lesions with adverse features. This is consistent with findings by Hubbeling et al., where bRT doses ≥30 Gy were associated with improved PFS on univariable analysis, but not on multivariable analysis when post-bRT LDH was incorporated, suggesting an implicit bias where higher doses were used for patients with lower disease burdens (23).

Although our sample size and limited number of events limited our ability to assess how cytoreduction may impact risk of CAR-T cell induced toxicity, including cytokine release syndrome and neurotoxicity, data also suggest that the associated cytoreduction with bRT may also benefit that outcome. In a systematic review by Al-Ibraheem et al., baseline disease burden measured by MTV, TLG, SUV_max_, and SUV_mean_ all were potentially associated with toxicity (28). Therefore, based on extrapolation from outcomes data, it is possible that, by cytoreducing pre-infusion disease extent, bRT may be able to reduce the morbidity of CAR-T cell infusion. This represents an important future next step in studies of how bRT may be able to benefit patients with R/R LBCL undergoing CAR-T cell therapy.

Our study has several limitations. First, although the intent behind this study was to examine cytoreduction attributed to bRT, 17.4% of patients received bCMT, which could impact both extent of cytoreduction and outcomes. We attempted to isolate bRT effects by comparing intra- and extra-radiation field reductions, finding out-of-field progression during bRT, which aligns with prior reports (16, 23). However, our limited patient numbers, single centric experience, and the inherent bias toward more aggressive therapy for extensive disease present challenges in attributing cytoreduction solely to bRT. Second, the retrospective nature of our study also introduces variability in the timing of PET scans relative to bRT, potentially affecting the accuracy of disease burden assessment and response. Additionally, there was also heterogeneity in patient characteristics, baseline disease characteristics, and radiation technique. Use of delta radiomics partially mitigates differences in baseline disease burden by focusing primarily on extent of cytoreduction, but, nevertheless, there will be inherent differences in prognoses based on baseline disease characteristics, including the role of delta radiomics in outcomes. Finally, despite these limitations and the small sample size, our findings corroborate the substantial cytoreductive effect of bRT and suggest that quantitative metrics of this reduction could prognosticate both local and overall disease control. We are currently conducting a prospective study to further investigate the standardized application of comprehensive bRT and subsequent imaging, including the role of delta radiomics in outcomes (NCT05800405).

## Conclusions

According to our results, delta-radiomic analysis revealed substantial reductions in MTV, SUV_max_, SUV_mean_, and TLG following bRT. Patients and treated lesions with greater changes in these PET metrics had superior outcomes. Further research should confirm the prognostic value of interim PET assessment following bRT and examine whether intensified bRT, via either systemic therapy (ST) or higher radiation therapy (RT) doses, impacts patient outcomes.

## Data Availability

The raw data supporting the conclusions of this article will be made available by the authors, without undue reservation.
