# Supplementary material for: Prognostic significance of fludeoxyglucose positron emission tomography delta radiomics following bridging therapy in patients with large B-cell lymphoma undergoing CAR T-cell therapy
Source: Front Immunol. 2024 Oct 1;15:1419788. doi: 10.3389/fimmu.2024.1419788 (PMC11473334; doi:10.3389/fimmu.2024.1419788)
Supplement: Supplementary file 1 [file DataSheet1.docx]

**SUPPLEMENT**

**Supplementary Figure 1: Quantification of changes in laboratory data**

**
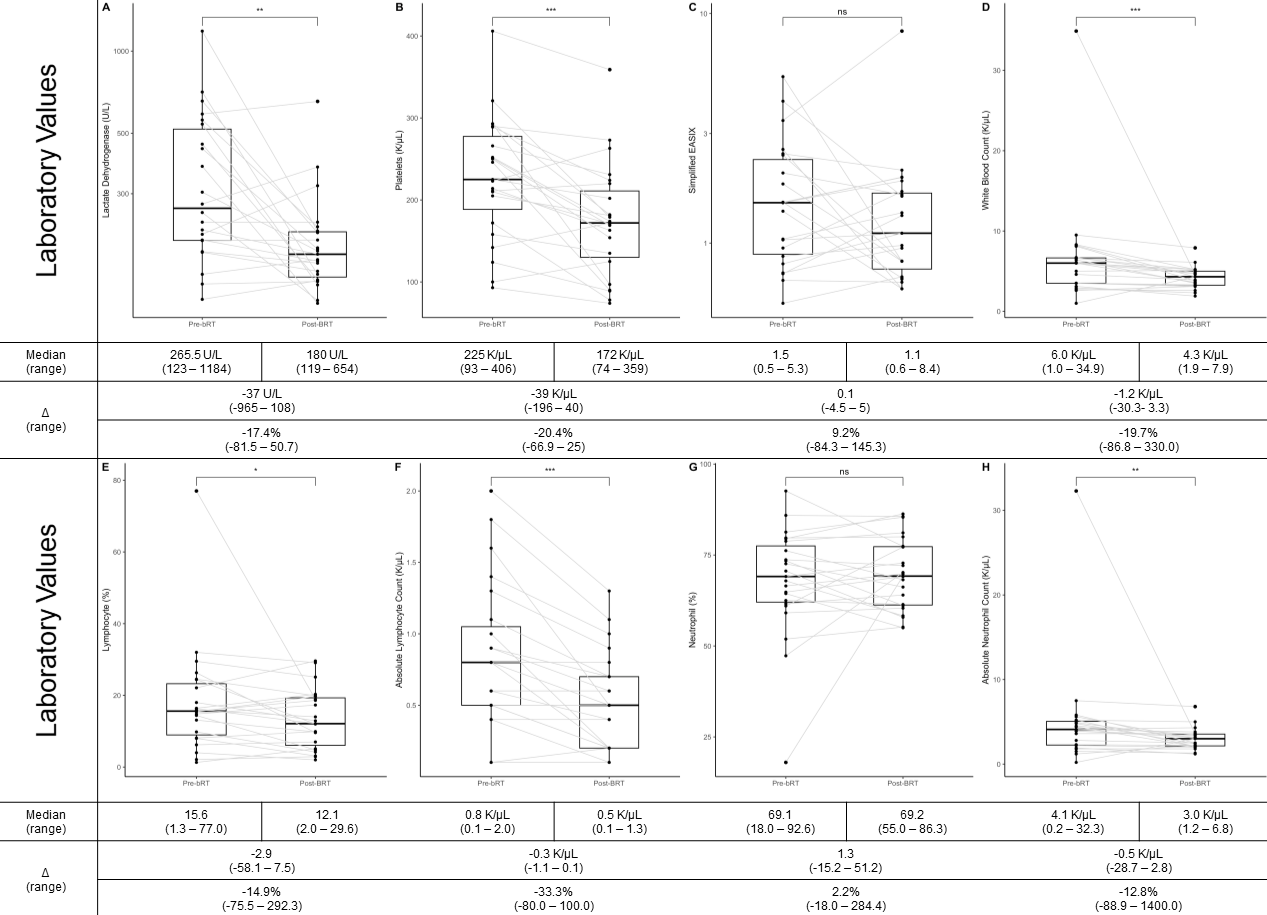
**

**Supplementary Figure 2: Dose-Response**

**
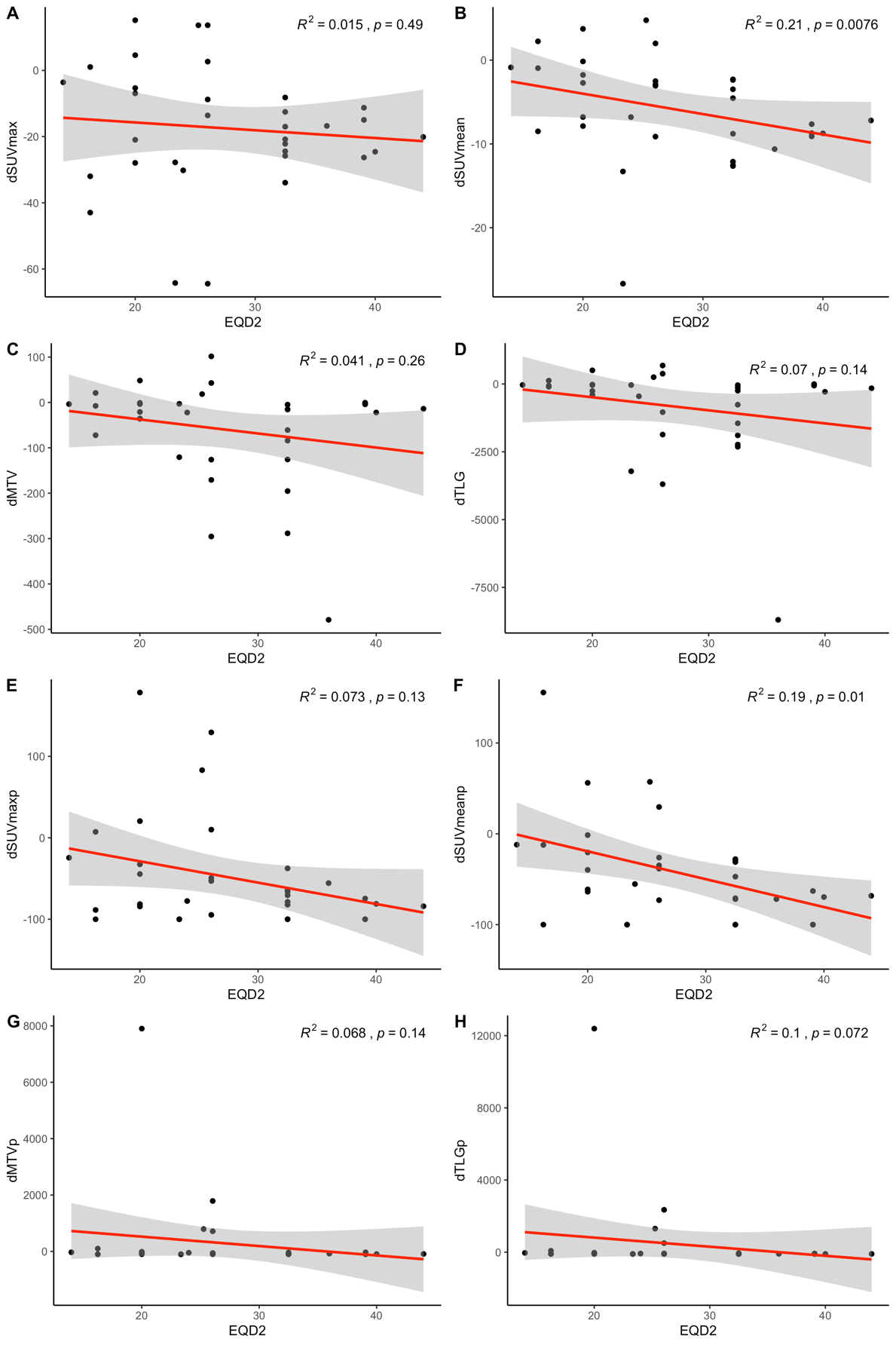
**

**Supplementary Figure 3: PFS**

**
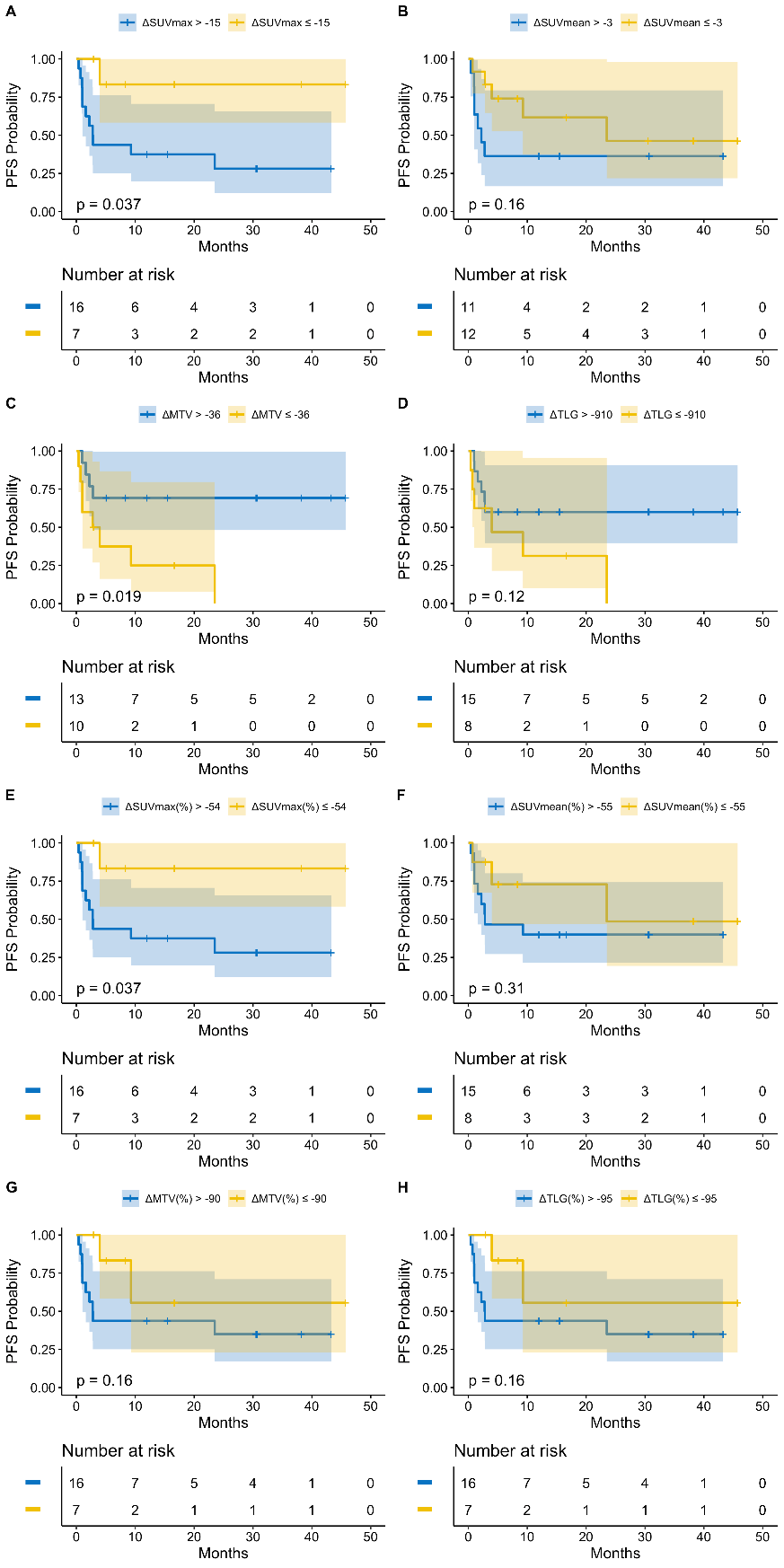
**

**Supplementary Figure 4: OS**

**
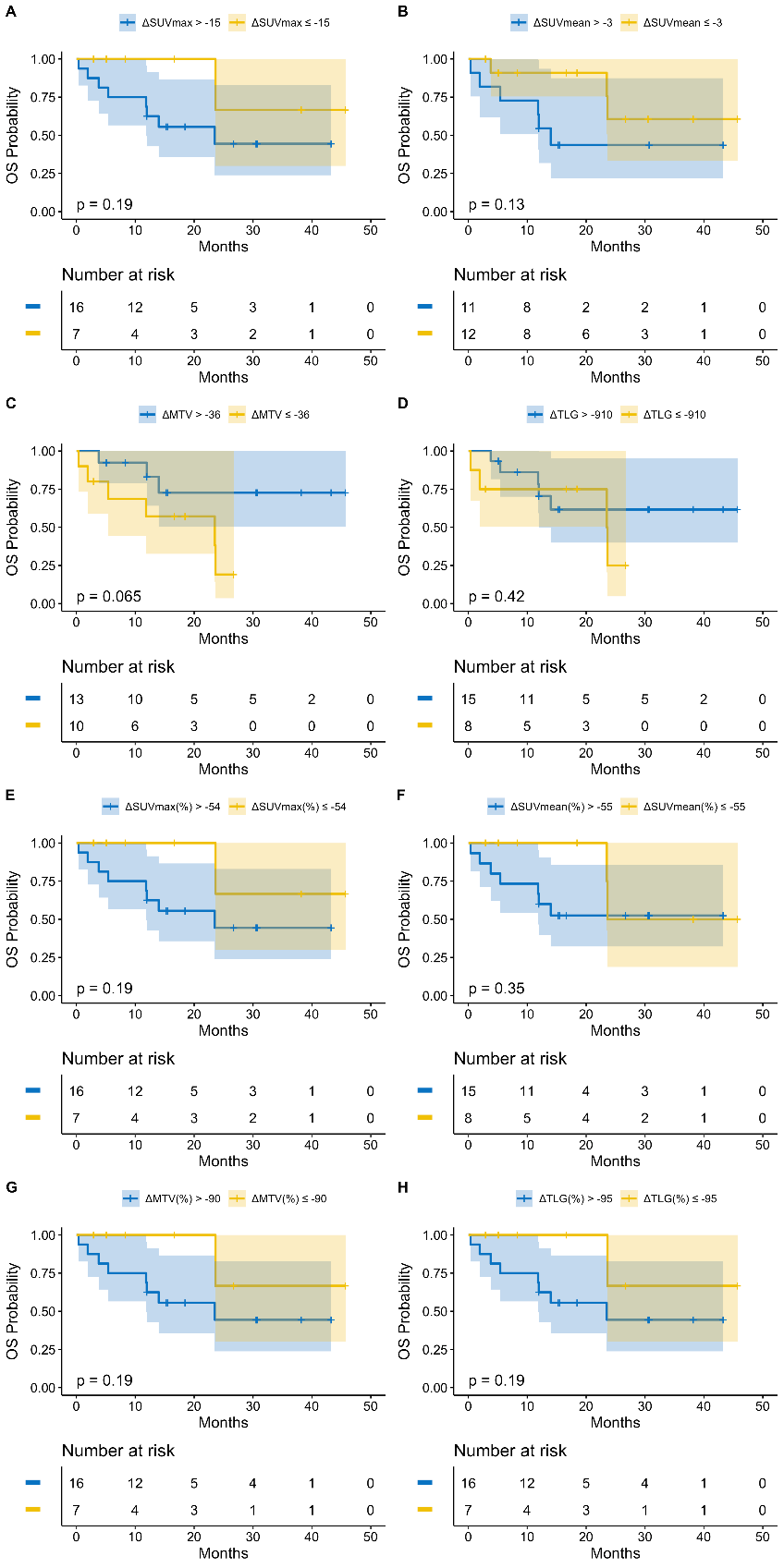
**

**Supplementary Figure 5: FFDP**

**
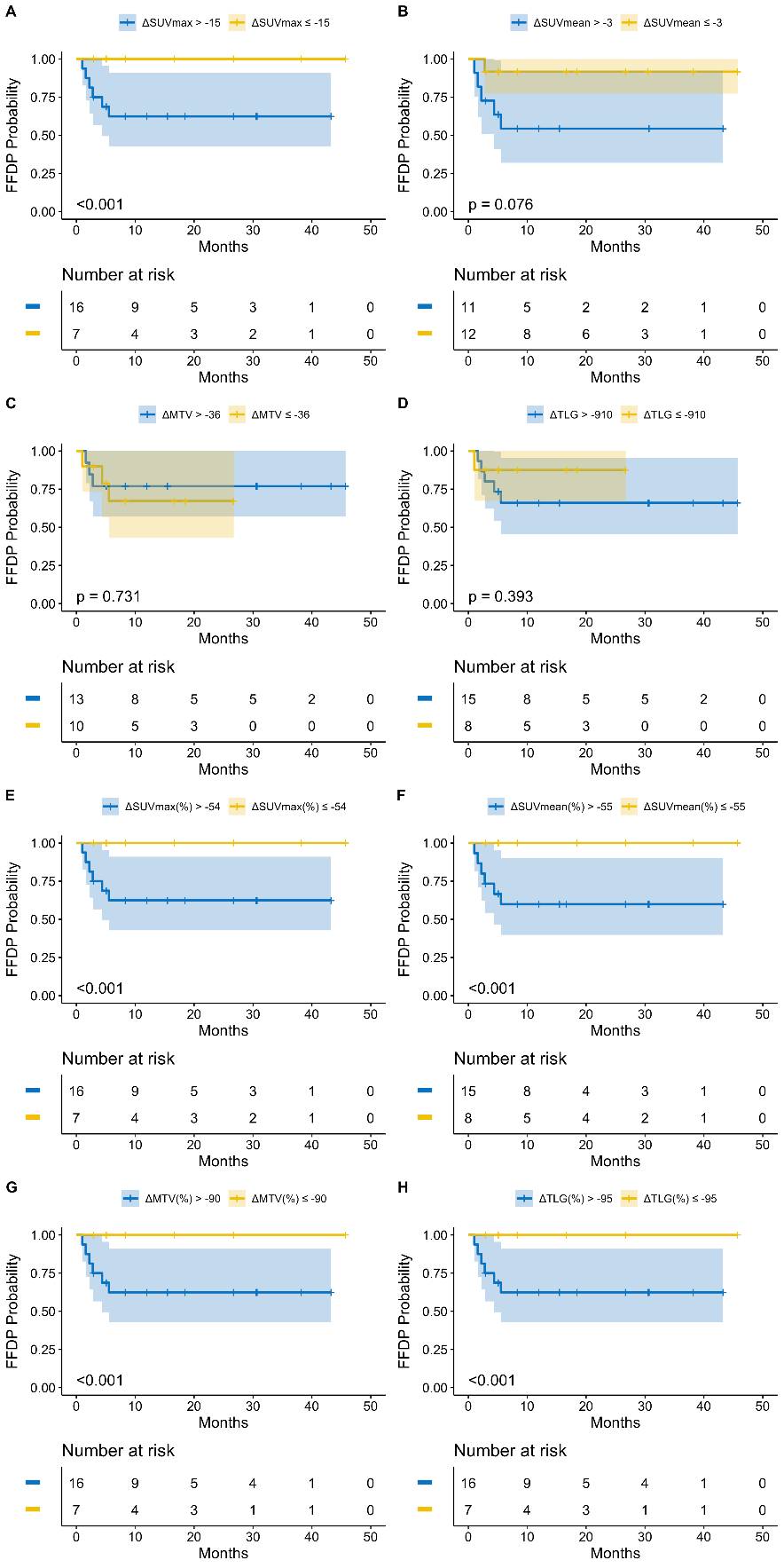
**

**Supplementary Figure 6: LC**

**
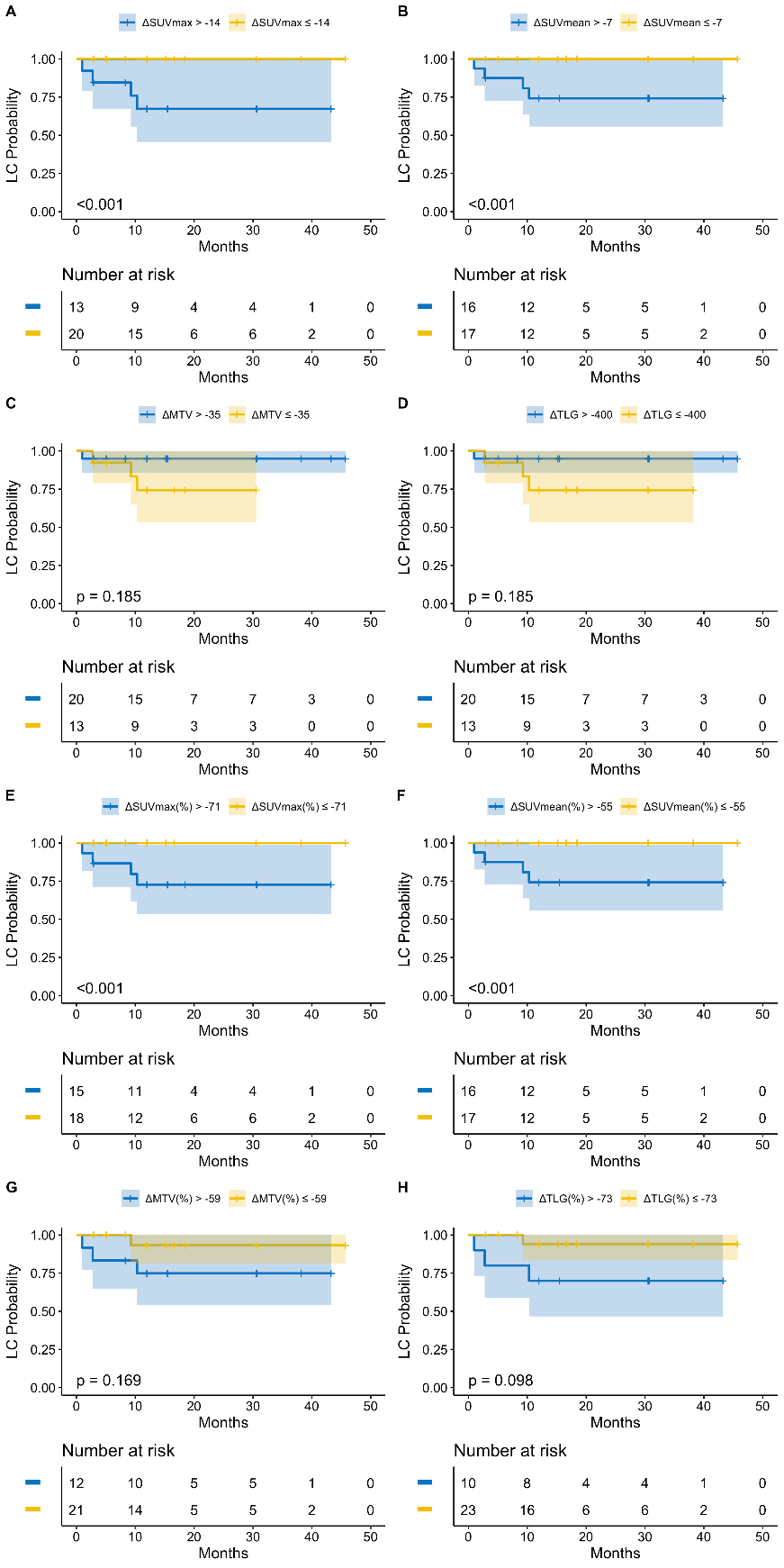
**

**Supplementary Figure 7: Dose Outcomes**

**
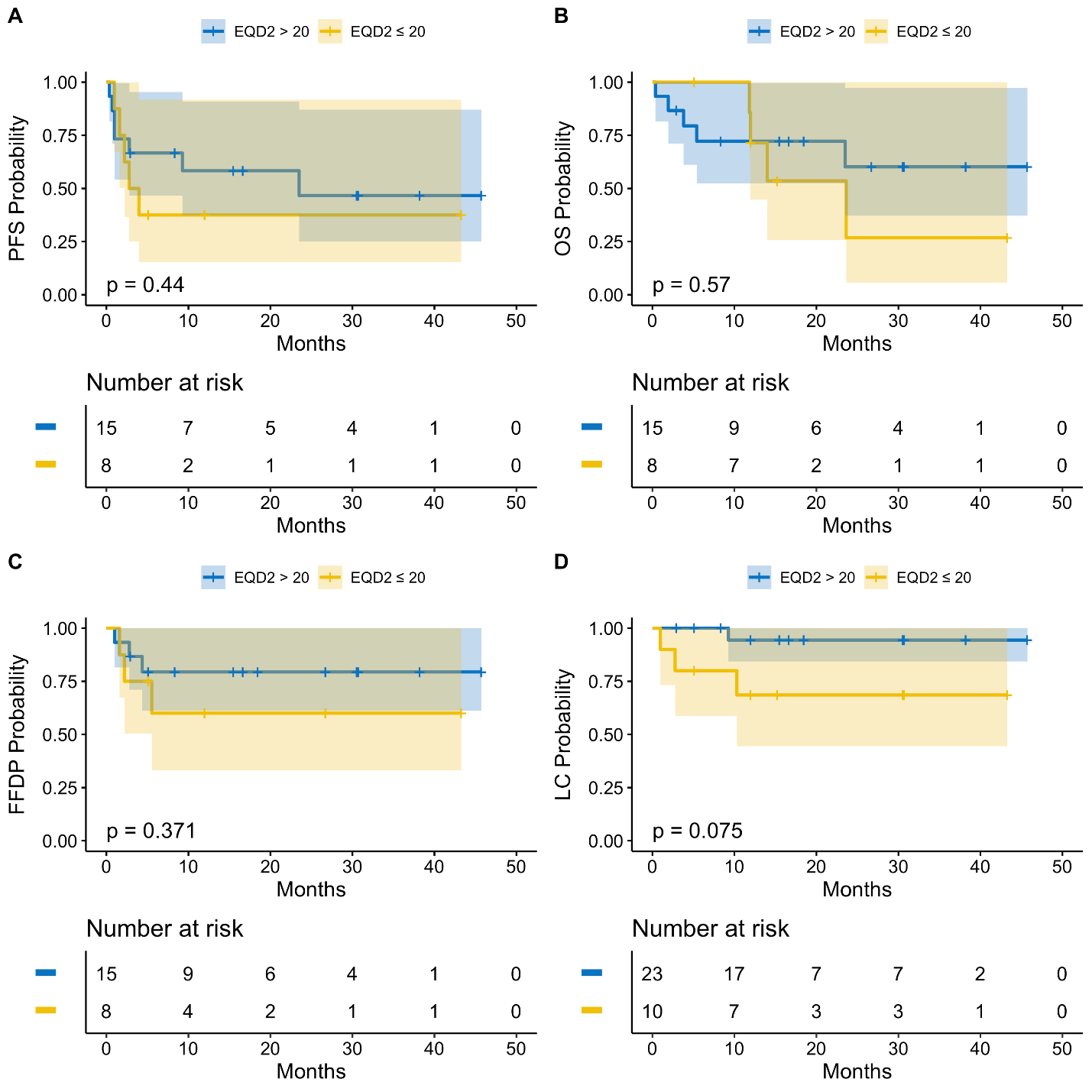
**
